# An atlas of inter- and intra-tumor heterogeneity of apoptosis competency in colorectal cancer tissue at single-cell resolution

**DOI:** 10.1038/s41418-021-00895-9

**Published:** 2021-11-09

**Authors:** Andreas Ulrich Lindner, Manuela Salvucci, Elizabeth McDonough, Sanghee Cho, Xanthi Stachtea, Emer P. O’Connell, Alex D. Corwin, Alberto Santamaria-Pang, Steven Carberry, Michael Fichtner, Sandra Van Schaeybroeck, Pierre Laurent-Puig, John P. Burke, Deborah A. McNamara, Mark Lawler, Anup Sood, John F. Graf, Markus Rehm, Philip D. Dunne, Daniel B. Longley, Fiona Ginty, Jochen H. M. Prehn

**Affiliations:** 1 Department of Physiology and Medical Physics, Royal College of Surgeons in Ireland University of Medicine and Health Sciences, 123 St. Stephen’s Green, Dublin 2, Ireland; 2 Centre of Systems Medicine, Royal College of Surgeons in Ireland University of Medicine and Health Sciences, 123 St. Stephen’s Green, Dublin 2, Ireland; 3 GE Research, Niskayuna, NY 12309 USA; 4 Centre for Cancer Research & Cell Biology, Queen’s University Belfast, 97 Lisburn Road, Belfast, BT9 7AE Northern Ireland UK; 5 Department of Surgery, Royal College of Surgeons in Ireland University of Medicine and Health Sciences, 123 St. Stephen’s Green, Dublin 2, Ireland; 6 Centre de Recherche des Cordeliers, INSERM, CNRS, Université de Paris, Sorbonne Université, USPC, Equipe labellisée Ligue Nationale Contre le Cancer, Paris, France; 7 Beaumont Hospital, Beaumont Road, Dublin 9, Ireland; 8 Institute of Cell Biology and Immunology, University of Stuttgart, Allmandring 31, 70569 Stuttgart, Germany

**Keywords:** Cancer microenvironment, Tumour heterogeneity

## Abstract

Cancer cells’ ability to inhibit apoptosis is key to malignant transformation and limits response to therapy. Here, we performed multiplexed immunofluorescence analysis on tissue microarrays with 373 cores from 168 patients, segmentation of 2.4 million individual cells, and quantification of 18 cell lineage and apoptosis proteins. We identified an enrichment for BCL2 in immune, and BAK, SMAC, and XIAP in cancer cells. Ordinary differential equation-based modeling of apoptosis sensitivity at single-cell resolution was conducted and an atlas of inter- and intra-tumor heterogeneity in apoptosis susceptibility generated. Systems modeling at single-cell resolution identified an enhanced sensitivity of cancer cells to mitochondrial permeabilization and executioner caspase activation compared to immune and stromal cells, but showed significant inter- and intra-tumor heterogeneity.

## Introduction

Alterations in apoptosis signaling is key step in tumorigenesis [1] and previous quantitative studies in solid tumor tissues found significant, but often complex differences in levels of individual anti- or pro-apoptotic proteins between different patients [2–5]. Predictions of individual patient’s apoptosis susceptibility are complicated by the signaling redundancies in key apoptosis pathways, as exemplified in the mitochondrial apoptosis pathway [6–8]. BH3-peptide profiling has been successfully applied to predict outcome and responses to cancer therapeutics in solid cancers; however, this technique requires fresh tissue [9]. Other groups, including our own, have used gene expression or protein level (western blotting and reverse protein phase array) data of apoptosis-regulating genes from fresh-frozen or formalin-fixed tissues as input into deterministic signaling network models to estimate the intrinsic apoptosis sensitivity of individual tumors. The ordinary differential equation-based models DR_MOMP [2] and APOPTO-CELL [10, 11] calculate a cell’s sensitivity to induce apoptosis as a two-step process with little feed-back from one to the other process [12]. DR_MOMP models the BCL2 signaling network triggered upon activation of BH3-only proteins, and APOPTO-CELL models the activation of caspase-3 downstream of mitochondrial outer membrane permeabilization (MOMP). Both models have been validated experimentally in colon and other cancer cells [2–5, 13–16].

Notwithstanding the successful application of these techniques in predicting chemotherapy responses and clinical outcome in cancer patients, these “bulk” techniques require a tissue homogenate to be analyzed and come with loss of important spatial information including the precise cell-of-origin of the signals. It is feasible that some tumors are more resistant to therapy than others by harboring resistant sub-populations, which is in line with evidence indicating the role of tumor heterogeneity in determining clinical outcome and responses to therapy [17, 18]. Such resistant cell populations could give rise to more aggressive tumors on recurrence. Similarly, chemo- or radiation therapy not only affects tumor cells, but also cells in the tumor microenvironment such as immune cells; therefore, a higher apoptosis sensitivity of anti-tumor immune cells compared to cancer epithelial cells may be detrimental to patients.

To describe the extent of inter-individual and intra-tumor heterogeneity in apoptosis signaling, herein we employed an innovative multiplexed immunofluorescence imaging technique (Cell DIVE™), which is comprised of a repeated stain-image-dye-inactivation sequence using direct antibody-fluorophore conjugates, as well as a small number of primary antibodies from distinct species with secondary antibody detection [19], followed by single-cell segmentation in a colorectal tumor tissue cohort. This enabled us to calculate each individual cell’s apoptosis sensitivity through single-cell systems modeling, and quantitatively describe inter- and intra-tumor heterogeneity of the mitochondrial apoptosis pathway among different cell types that constitute a colorectal tumor.

## Results

**Fig. 1:**
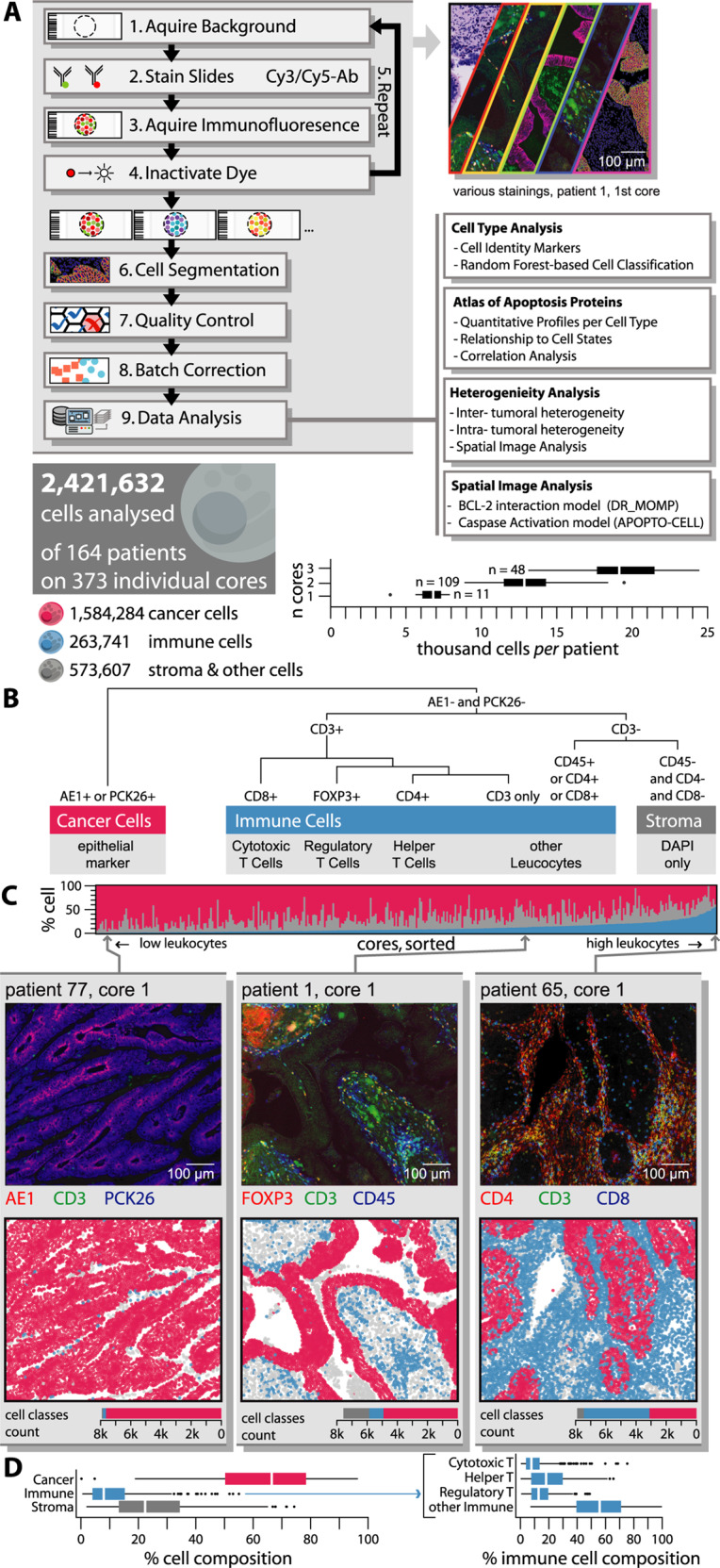
Overall workflow for image processing, data generation and analysis. **A** Simplified workflow of the Cell DIVE™ platform and data analysis. In total, we quantified 18 proteins in a total of 2.4 million cells in 373 tissue microarray (TMA) cores from 168 patients (12 stage II, 132 stage III, 9 stage IV). On average, we found 6492 (SD 1228) cells per core; totaling on average 14,414 (SD 4196) cells per patient (1–3 cores; F). **B** Random forest was used to differentiate cells using DAPI, and epithelial and CD markers. **C** The majority of cores consisted of epithelial like cancer and stroma cells, **D** with less than 20% of cells being immune cells in the majority of cores (ANOVA, Tukey post-hoc).

To explore the levels of key proteins of the mitochondrial apoptosis pathways in colorectal cancer (CRC) tissue at the single-cell level, we performed Cell DIVE™ multiplexing of nine pro- and anti-apoptotic proteins in regions of resected primary tumors in 355 tumor cores derived from 168 stage III CRC patients (Fig. 1A). Apoptosis signaling protein selected for analysis included BCL2, BCL(X)L, MCL1, BAK, and BAX that regulate the process of MOMP, as well as PRO-CASPASE 9, PRO-CASPASE 3, XIAP, and SMAC (DIABLO) that control the process of executioner caspase activation downstream of MOMP. These nine proteins were used as input variables for our deterministic models DR_MOMP [2] and APOPTO-CELL [10, 11] to calculate the apoptosis sensitivity markers “sensitivity for MOMP” and “caspase activity.” Additional proteins selected for this study included cell identity markers (CD3, CD4, CD8, CD45, FOXP3, PCK26, and cytokeratin AE1), as well as proteins used for cell segmentation analysis (Na+/K+-ATPase, cytokeratin AE1, PCK26, and S6).

Cells were classified into different cell types based on cell identity markers for cancer/epithelial cells (positive for cytokeratin AE1 or PCK26), immune cells (positive for CD3, CD4, CD8, or CD45), and other stromal cells negative for any of these markers. For more extensive cell classification, a Random Forest model was trained with 15,184 manual annotated cells (0.6% of total cells) and CD3, CD4, CD8, CD45, and FOXP3 levels, and applied on 99.9% of all cells to further differentiate immune cells into cytotoxic, regulatory, helper T, and other immune cells (Fig. 1B). The model classified 65.7% as (epithelial like) cancer cells (type II error 3.0% in training set), 23.6% other stromal cells (type II error 8.1%), and 10.7% as immune cells (type II error 3.0%), of which 2.0% were helper (type II error 28.8%), 1.4% regulatory (type II error 7.4%), 1.3% cytotoxic (type II error 28.0%), and 6.0% other T or immune cells (type II error 18.8%). The cell type composition in CRC core tissues varied significantly, with some cores showing predominantly cancerous/epithelial cells in the absence of immune cell infiltration, and others showing very high levels (up to 55%) of immune cells (Fig. 1C, D). A bootstrap analysis with randomly sampled pairings found cell type composition in tumors of patients with paired-cores, despite high heterogeneity, more similar to each other compared to random pairings (Supplementary Fig. 1), suggesting that cell type composition was a biological feature of individual tumors.

### Tumor cell atlas shows heterogeneous and cell type-specific enrichment of key proteins of the mitochondrial apoptosis pathway

**Fig. 2:**
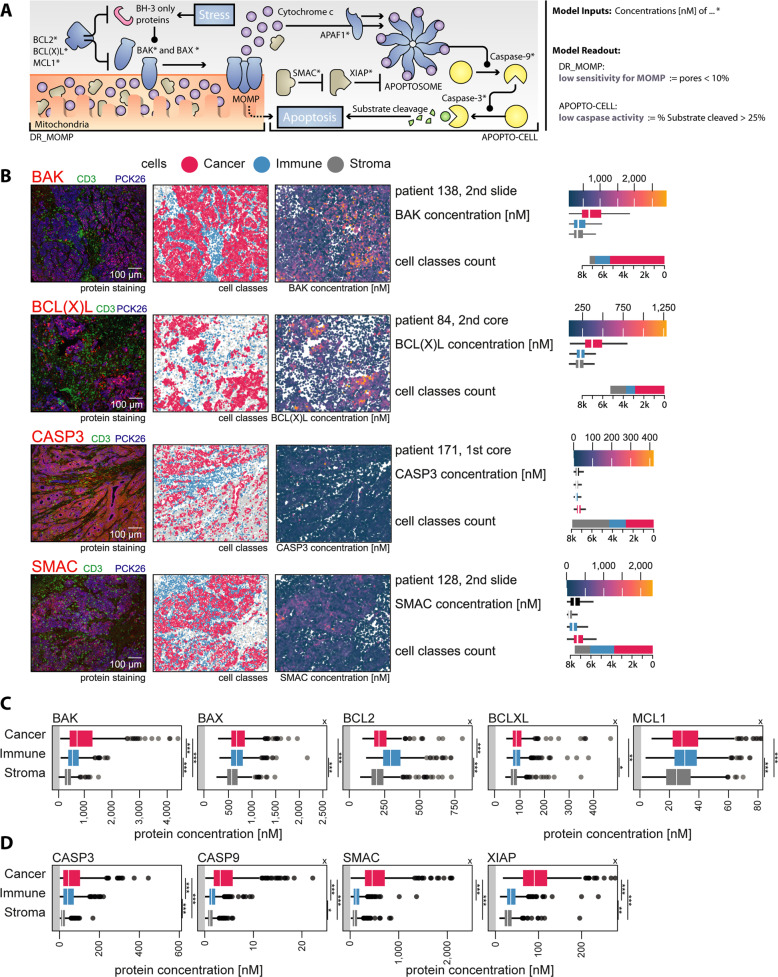
Difference in apoptosis protein levels between cancer, immune and stroma cells. **A** Graphical illustration of the modeled BCL2 pathway (DR_MOMP) upstream of MOMP and the modeled caspase pathway (APOPTO-CELL) downstream of MOMP **B** with four examples of the pre-batch-corrected protein staining, cell type classification, and calculated batch-corrected cell protein concentrations. Protein analysis of apoptosis proteins relevant for **C** the DR_MOMP model upstream of MOMP and **D** the APOPTO-CELL model downstream of MOMP in 373 cores. To determine the difference between protein quantification based on cell masks and quantification using the whole image, we first determined the median protein concentration of each core, stratified for cancer (red), immune (blue), and stroma (gray) cells (ANOVA, Tukey post-hoc). *x* marks panels with cropped high value outliers.

We next calculated molar protein profiles for key proteins controlling apoptosis (Fig. 2A, B) and used as input for the deterministic systems models, DR_MOMP (Fig. 2C) and APOPTO-CELL (Fig. 2D). Analysis of five key BCL2 proteins that control the process of MOMP demonstrated a significant enrichment in anti-apoptotic BCL2 in immune cells when compared to cancerous epithelial cells or other stromal cells, while anti-apoptotic BCL(X)L and MCL1, although statistically enriched in cancer epithelial cells, were more homogenously distributed between the three cell types (Fig. 2C). Mean levels of MCL1 were in general lower compared to BCL2 and BCL(X)L, confirming previous studies [2]. Of note, pro-apoptotic BAK showed a strong enrichment in cancer cells (Fig. 2C). BAX, although statistically enriched in cancer cells, was more homogenously distributed between the three cell types (Fig. 2C).

Proteins controlling executioner caspase activation downstream of MOMP also showed a heterogeneous distribution between cell types, with XIAP, SMAC, PRO-CASPASE 3, and PRO-CASPASE 9, all at higher levels in cancer cells when compared to immune cells (Fig. 2D). Stromal cells showed the lowest levels of these proteins, suggesting that the apoptotic machinery downstream of MOMP is suppressed in non-transformed cells when compared to cancer epithelial cells.

**Fig. 3:**
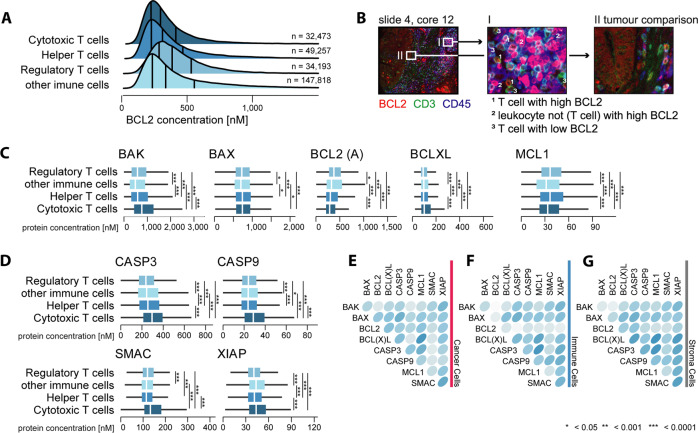
Single-cell analysis of apoptosis protein levels in immune cells. Global immune single-cell protein analysis of apoptosis proteins relevant for **A**–**C** the DR_MOMP model upstream of MOMP and **D** the APOPTO-CELL model downstream of MOMP (ANOVA, Tukey post-hoc) in 373 cores. **B** Virtual IHC staining with BCL2 (red), CD3 (green), and CD45 (blue) shows that BCL2 level vary largely between immune cells. **E**–**G** Calculated median spearman correlation coefficient between proteins, stratified for **E** cancer, **F** immune, and **G** stroma cells. A more detailed correlation plot, including inter quantile ranges, is provided as Supplementary Fig. 4.

Investigating apoptosis proteins within immune cells, we found higher levels of BAK, XIAP, SMAC, PRO-CASPASE 3, and PRO-CASPASE 9 and lower levels of BCL2 in cytotoxic T cells when compared to helper or regulatory T cells (Fig. 3A–D). These findings suggest that cytotoxic T cells may represent T cells most sensitive to the activation of mitochondrial apoptosis.

Utilizing transcriptional data derived from flow-sorted immune (*n* = 6), epithelial (*n* = 6), and fibroblast (*n* = 6) populations isolated from CRC primary tumor tissue (GSE3939625; Supplementary Table 3), we likewise identified elevated levels of bcl2 mRNA levels in leukocytes compared to cancer (epithelial) cells (analysis of variance [ANOVA] *p* = 0.006, Tukey post-hoc *p* = 0.005). We also identified higher levels of *bax* and *mcl1* mRNA levels in leukocytes compared to cancer cells, and in other stromal cells (fibroblasts) compared to cancer cells (ANOVA *p* ≤ 0.01, Tukey post-hoc *p* < 0.01; Supplementary Fig. 2). We did not find any significant differences in mRNA levels of *bak1*, *bcl2l1* (BCL(X)L), and caspases. While these data “validated” the enrichment of Bcl-2 in immune cells, they also suggested that mRNA levels may not accurately reflect protein level data when investigating individual cell populations.

Calculating the cores’ quartile coefficients of dispersion (COD; Supplementary Fig. 3), a measure of the spread of the protein levels, we found that immune cells had a greater COD for BCL2 and BAK compared to cancer and stroma cells. Stroma cells showed the highest, and cancer epithelial cells the lowest, COD for MCL1, APAF1, and PRO-CASPASE 3. Cancer cells showed greater CODs of SMAC compared to immune and stroma cells.

Single-cell correlation analysis (Fig. 3E, F) of 1,556,581 cancer cells demonstrated high, positive median Spearman’s correlation coefficients (*ρ* > 0.5) between BAK and BAX levels. Levels between BAK (and BAX) and PRO-CASPASE 3 (and PRO-CASPASE 9), BCL(X)L and BCL2, PRO-CASPASE 3 and BCL2, BCL2 and MCL1, BCL2 and XIAP, SMAC and BCL(X)L, PRO-CASPASE 3 and PRO-CAPSASE 9, and PRO-CASPASE 3 and XIAP had high positive median correlation coefficients in cancer and stromal, but not immune cells. Spearman’s correlation coefficient between BCL(X)L and MCL1 and XIAP, and SMAC and XIAP levels was >0.5 in all cells. Generally, correlations between individual proteins were similar between leukocytes and stromal cells and frequently differed from those in cancer cells, validating at the single-cell level that transformed cells deviate from a physiological regulation of apoptosis.

### Single-cell systems modeling of apoptosis sensitivity shows inter-individual differences in apoptosis sensitivity and an enhanced ability of tumor cells to undergo Caspase-3-dependent mitochondrial apoptosis

**Fig. 4:**
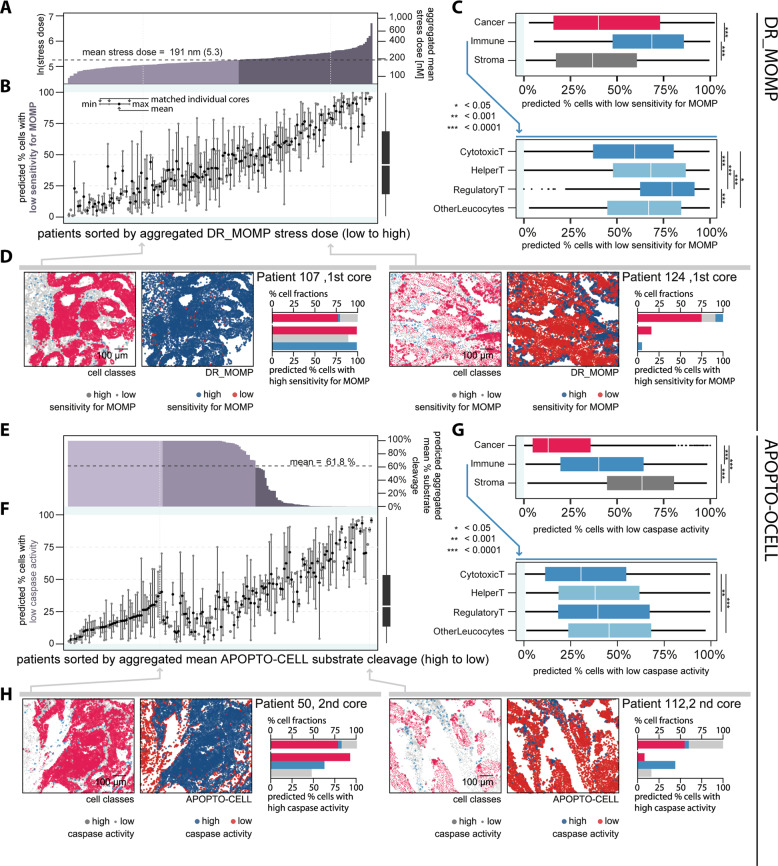
Cancer cells show a higher capability to activate caspase signaling compared to immune and stromal cells. Results of the cell-by-cell analysis using the apoptosis models DR_MOMP [2] and APOPTO-CELL [10, 11] in 373 cores. We first analyzed **A**–**D** DR_MOMP and subsequently **E**–**H** APOPTO-CELL. **A**, **E** First we determined model predictions of required stress to induce MOMP (DR_MOMP) and % substrate cleavage upon MOMP (APOPTO-CELL) based on aggregated mean protein level for each patient, using the pool of all cells of multiple cores. Subsequently, we calculated the cores’ cell fractions with **B** high/low sensitivity for MOMP (DR_MOMP) and **F** high/low substrate cleavage (APOPTO-CELL) using individual cell protein levels. We compared cores’ fractions with high/low **C** sensitivity for MOMP and **G** caspase activity stratified for cancer (red), immune (blue), and stroma (gray) cells (ANOVA, Tukey post-hoc).The panels **D** and **H** show examples of individual cores with high/low **D** sensitivity for MOMP and **H** caspase activity. In **A**, **B** and **E**, **F**, cores were sorted from high apoptosis sensitivity (left) to low apoptosis sensitivity (right), respectively.

Using quantitative Bcl-2 protein profiles of BAK, BAX, BCL2, BCL(X)L, and MCL1 as model input for DR_MOMP, we were able to calculate the sensitivity of individual tumor cells to the process of mitochondrial apoptosis initiation (Fig. 4A, B). We found significant differences in % cells with low sensitivity for MOMP in this otherwise homogeneous cohort of stage III CRC patients (Fig. 4B). Between patient-matched cores, we found a mean difference of 18.8% ± SD 14.1% and a mean SD of 14.0% cells with low sensitivity for MOMP. When stratifying DR_MOMP calculations for individual cell types, we found that, on average, significantly fewer cancer cells and stromal cells exhibited low sensitivity for MOMP when compared to immune cells (Fig. 4C, upper). Among immune cells, regulatory T cells were found to have largest population of single cells with low sensitivity for MOMP (Fig. 4C lower). In line with our analysis on protein level (Fig. 3), cytotoxic T cells were overall significantly more susceptible to apoptosis stimuli compared to other immune cells

When investigating the sensitivity of individual tumor cells to undergo caspase 3 activation (once the process of MOMP is activated) using the APOPTO-CELL systems model downstream of MOMP, we similarly found significant differences between individual patients (Fig. 4E) and cores (Fig. 4F). Between patient-matched cores we found a mean difference of 18.8% ± SD 15.7% and a mean SD of 13.8% cells with low predicted caspase activity. Importantly, when investigating individual cell types, we found that cancer cells were predicted to show a higher caspase activity compared to immune cells and stromal cells, with latter showing the greatest fraction of cells with low predicted caspase activity (Fig. 4G).

**Fig. 5:**
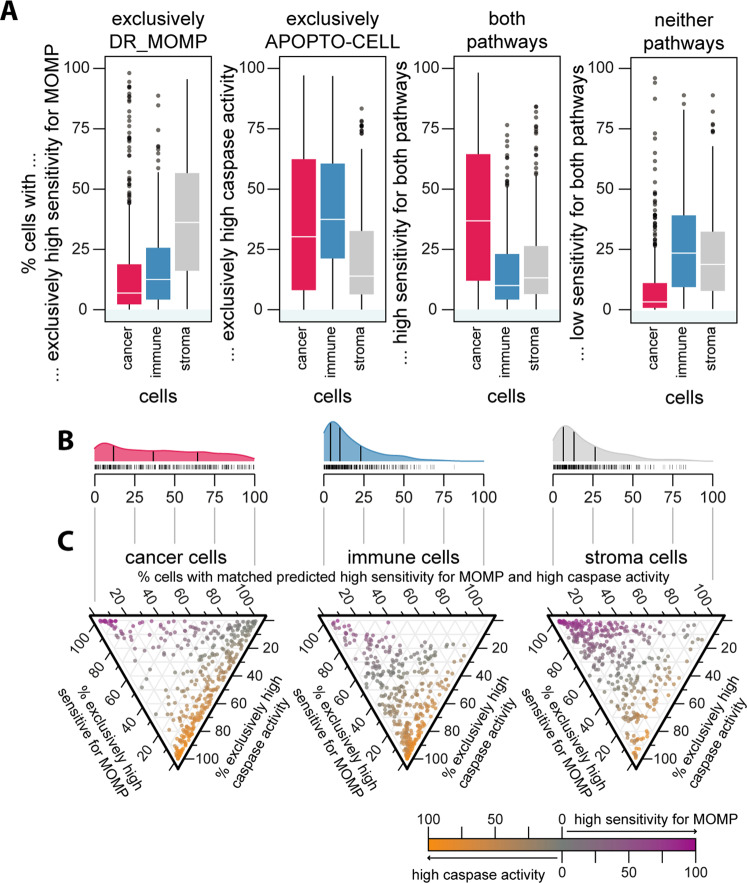
Cancer cells are sensitive for both apoptosis pathways. We determined cores’ cells that **A** exclusively showed high sensitivity for MOMP (left), high caspase activity, high responses in both apoptosis pathways, and low responses in both apoptosis pathways (right; ANOVA, Tukey post-hoc). **B**, **C** Ternary plot of individual core’s cell fraction for exclusively pathway responses or sensitivity in both pathways. Overall, cancer cells show high sensitivity for the DR_MOMP modeled BCL2 pathway upstream of MOMP with about half showing also high caspase activity modeled by APOPTO-CELL. Stroma cells showed exclusively high sensitivity for the apoptosis pathway upstream for MOMP, while immune cells showed exclusively high sensitivity for MOMP.

The activation of mitochondrial (or intrinsic) apoptosis is considered to be a two-step process, with little feedback from one to the other process. Assessing apoptosis sensitivity up- and downstream of MOMP showed that cancer cells are sensitive for both apoptosis pathways in the majority of tumors and that only a small fraction of cores showed low sensitivity in both pathways at the same time (Fig. 5A). In contrast, immune and stroma cells had a higher fraction of cells that showed low sensitivity in both pathways, and a lower fraction of cells that showed high sensitivity in both, compared to cancer cells (Fig. 5A, B). Of the cancer cells that show low sensitivity in one and high sensitivity in the other pathway, we found that a majority of cancer cells showed a low MOMP sensitivity and a predicted high caspase activity (Fig. 5A, C). In contrast, the majority of immune cells showed a predicted high caspase activity but a low sensitivity for MOMP, and the majority of stroma cells showed a high sensitivity for MOMP but a predicted low caspase activity (Fig. 5A, C). Collectively, the data suggested that the majority of cancer cells showed a high sensitivity for at least one of the two apoptosis pathways, and that cancer cells were overall more likely to respond to both signaling pathways when compared to immune or stromal cells.

### Analysis of intra-tumoral heterogeneity

**Fig. 6:**
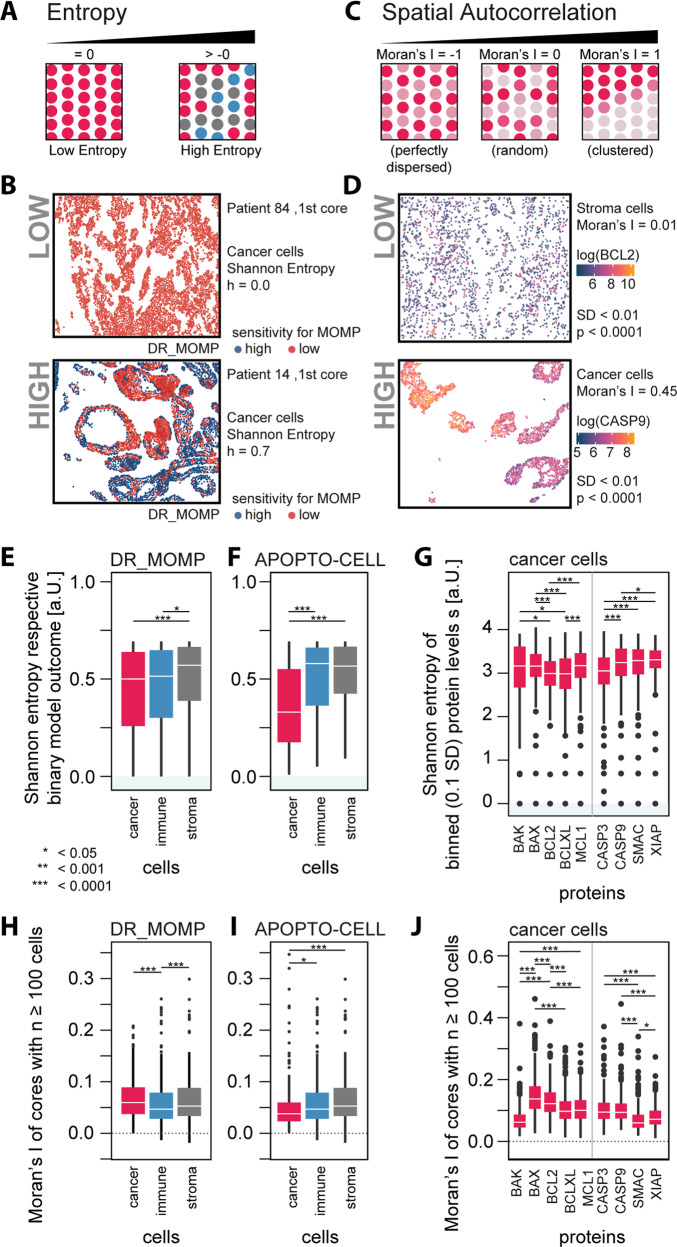
Heterogeneity analysis calculating cells’ (A, B, E–G) entropy and (C, D, H–J) Moran’s I for apoptosis model predictions as well protein levels. **A** Entropy (information theory) is a measurement for the bias to one state, with low entropy marking captaincy for a one state and high entropy marking uncertainty for one or multiple states. **C** Moran’s I is a measurement of spatial autocorrelation with Moran’s I approaching 0 and <0 indicating spatial dispersion and Moran’s I approaching 1 marking spatial clustering. Only cores with cell population ≥100 were considered. Panels **B** and **D** show examples of low and high entropy and Moran’s Is. We determined the binary Shannon entropy for **E** low/high sensitivity for MOMP (DR_MOMP) and **F** low/high caspase activity (APOPTO-CELL; ANOVA, Tukey post-hoc), finding surprisingly significant lower entropy in cancer cells (red) compared to immune (blue) and stroma cells (gray). **G** Subsequently, we calculated the Shannon Entropy for the proteins using bins for protein level with a bin width of *z*-score = 0.1 SD for each protein, respectively. The calculated Shannon Entropy for stroma and Immune cells can be found in Supplementary Fig. 5. Next, we determined cores’ Moran’s I for low/high **H** sensitivity for MOMP, **I** caspase activity, and **J** respective protein levels (in cancer cells). Calculated Moran’s I for Stroma and Immune cells can be found in Supplementary Fig. 6. **J** While Moran’s I around 0 shows no spatial autocorrelation, values around 0.2 or greater indicate presence of local spatial autocorrelation within the cores.

While investigating apoptosis sensitivity at the single-cell level using our systems models, we also noticed that certain patients showed a significant intra-tumor heterogeneity among cancer cells, while other patients showed a more homogenous distribution in model predictions (Fig. 4B, F). To assess intra-tumoral heterogeneity, we assessed the Shannon Entropy (Fig. 6A, B, E–G) and Moran’s I (Fig. 6C, D, H–J) of the apoptosis model output and measured protein levels. A low entropy, close to zero, suggests homogenous model predictions among all cells, which could either indicate systemic sensitivity or systemic resistance and are in contrast to heterogeneous cell states that display a high entropy (Fig. 6A). Moran’s I measures spatial autocorrelation, an estimate for spatial separation of cell states across cores. Moran’s I of 1 indicates a perfect spatial separation (e.g., left versus right separation), while values between 0 and –1 indicate random or perfect dispersion (checkerboard pattern), respectively (Fig. 6C).

While the difference was small, cancer cells and immune cells had significantly lower entropy compared to stroma cells for DR_MOMP (Fig. 6E). We found similar results for the predictions of APOPTO-CELL, but with the difference between cancer and immune cells much more distinct (Fig. 6F), which is in line with the overall calculated high caspase activity among cancer cells (Fig. 5A, C). We did not find any statistically significant difference in Moran’s I between the different apoptosis models (Fig. 6H, I). Although minor, cores’ Moran’s I of immune cells were lower compared to cancer cells (ANOVA and Tukey post-hoc, Fig. 6H) and cancer cells with different predictions for APOPTO-CELL were significantly more randomly dispersed compared to stromal cells (ANOVA and Tukey post-hoc; Fig. 6I).

Studying the entropy of protein levels using binned protein levels (normalized bin size = 0.1 SD) in cancer cells (Fig. 6G), we found significant lower entropy in BCL2, BCL(X)L, and PRO-CASPASE 3 compared to other proteins in these cells. Compared to immune and stroma cells, cancer cells showed higher entropy in levels of BAK, BAX, PRO-CASPASE 9, SMAC, and XIAP (ANOVA *p* < 0.05, Tukey post-hoc *p* < 0.05; Supplementary Fig. 5).

Calculating Moran’s I for cells’ protein levels, we found that the majority of cancer cells have a score less than 0.2 suggesting a tendency toward a low correlation between protein level and the distance between cells (Fig. 6J). Among the proteins relevant for DR_MOMP, BAX and BCL2 showed the higher Moran’s I compared to BAX, BCL(X)L, and MCL1. Among proteins used in the APOPTO-CELL model, SMAC had the lowest Moran’s I compared to PRO-CASPASE 3, 9, and XIAP.

Of note, since immune cells are more mobile than epithelial or stroma cells, we would assume to find the lowest Moran’s I in these cells. However, this was only the case for BAK, BCL2, PRO-CASPASE 3, and GLUT1 (Supplementary Fig. 6). While numerically different, overall Moran’s I was similar for BAX, BCL(X)L, MCL1, SMAC, and CA9 if stratified for cell types. We observed the greatest difference between cells of different types for BAK, BCL2, GLUT1, HLA-I, and KI67 (Supplementary Fig. 6).

We also performed a Cox proportional hazard regression analysis to explore whether intra-tumoral heterogeneity in Caspase-3 activating proteins or APOPTO-CELL model output may influence disease-free survival (DFS) of patients. A total of 36 stage III patients were analyzed for which three cores each were available (*n* = 108 cores). Mean protein levels and model calculations in combination with scores for Entropy or Moran’s I were included in the analysis and tested for interaction (adjusted for patients’ age and sex; Supplementary Fig. 7 and Supplementary Table 4). In agreement with previous reports [3, 4, 20], individual mean protein levels were not consistently prognostic, while a predicted high caspase activity (high APOPTO-CELL model output) was associated with increased DFS when adjusted for either Shannon Entropy or Moran’s I (HR 0.6; 95% CI 0.5–0.8; *p* < 0.001). Shannon Entropy or Moran’s I of individual proteins or APOPTO-CELL model output was not prognostic. However, we found a significant interaction (HR 0.1; 95% CI 0.01–0.5; *p* < 0.01) between a high Moran’s I and the mean PRO-CASPASE 3 level with a HR 2.9 (95% CI 0.4–21.5, *p* = 0.3).

## Discussion

The present study constitutes the first report describing the quantitative and spatial distribution of key mitochondrial apoptosis proteins at single-cell resolution in intact cancer tissue. Using multiplexed immunofluorescence imaging we provide information on 2.4 million apoptosis protein profiles in six different cell types and deliver the first atlas of apoptosis signaling proteins in a large cohort of patients (168 CRC patients). We furthermore conducted a systems-based analysis of each individual cell’s apoptosis sensitivity. Our dynamic systems modeling estimated that cancer cells were generally more sensitive to apoptosis signaling than immune or stromal cells, however, with significant heterogeneity between patients.

A surprising observation was that, based on model predictions, the enhanced ability of cancer cells to activate apoptosis resulted from an enhanced ability to overcome both apoptosis barriers, MOMP and caspase-3 activation downstream of MOMP. This is interesting in the context that apoptosis resistance has been proposed as a key hallmark of many cancer cells [1]. Immune cells lacked sensitivity for MOMP due to their relatively high expression of BCL2. Stromal cells showed less sensitivity to caspase-3 activation (Fig. 6A, C). Enhanced “priming” of cancer cells to undergo MOMP compared to non-transformed cells has previously been demonstrated by BH3 profiling by Letai et al. [9, 21]. Interestingly, we also found that cytotoxic (CD8+) T cells were overall significantly more susceptible to apoptosis stimuli compared to other immune cells. This may be clinically relevant as tumor infiltration by cytotoxic T cells has been found to be positively correlated with survival in CRC [22]. Similarly, a low density of cytotoxic T Cells in tumor tissue after chemotherapy was associated with poor response in patients with rectal cancer [23]. Therefore, increased risk of apoptosis of cytotoxic T cells may abrogate benefits of chemo- or radiotherapy.

Interestingly, we found a strong enrichment of BCL-2 protein levels in immune cells when compared to cancer and other stromal cells. This finding may have implications regarding the possible use of BCL2 antagonists for the treatment of CRC, and BCL2 mRNA or protein levels as stratification tool for such therapy. Medema et al. also demonstrated an enrichment of BCL-2 in immune cells, and a limited window of BCL-2 reliance in CRC cells during disease progression [24]. Their study also demonstrated that expression of BCL(X)L, but not BCL2 or MCL1, correlated with outcome in chemotherapy-treated CRC patients. Furthermore, we found that MCL1 levels were enriched in epithelial cancer and immune cells, with significantly lower levels in stroma tissue. As MCL1 antagonists are also being currently developed as apoptosis sensitizers for MCL1-dependent cells, effects of MCL1 antagonists on immune cells may also need to be considered. Another interesting aspect of our study was the strong enrichment of BAK in cancer cells. Recently, agents have been developed that activate BAX and BAK directly [25], including molecules that do not interact with the BH3-binding pocket of anti-apoptotic proteins or pro-apoptotic BAK and induces cell death in a BAX-dependent fashion [26, 27]. Our results suggest that BAK in particular may represent an excellent target in CRC.

We also investigated intra-tumor heterogeneity in apoptosis signaling. While we did observe significant inter-individual and intra-tumoral heterogeneity in apoptosis sensitivity, our entropy and spatial image analyses of the mitochondrial apoptosis pathway did not suggest that cancer cells showed a set difference in cell-to-cell or spatial heterogeneity when compared to immune or other stromal cells. This suggests that such heterogeneity represents an intrinsic, non-genomic property not increased by the process of malignant transformation. This observation is supported by earlier studies in cell lines that demonstrated the importance of non-genomic heterogeneity in apoptosis signaling due to fluctuations in protein levels over the lifetime of a cell [28, 29]. Our exploratory analysis indicated that variables describing heterogeneity within some measured proteins may contribute to clinical responses of cancer patients (Supplementary Fig. 7). However, further in-depth analyses of intra-tumoral heterogeneity in larger patient cohorts with an extended list of heterogeneity measures in tumor cores and margins are required.

While we considered the levels of nine apoptosis markers, we did not take into account their protein state (such as BCL2 phosphorylation status [30]) or their intracellular localization. For example, the localization of BAX at mitochondria or in the cytosol was reported to be clinically relevant in acute myeloid leukemia [31] and hepatocellular carcinoma [32]. Similarly, we currently do not account for variations in levels of pro-apoptotic BH3 proteins or possible variation of cells in producing BH3 proteins in response to genotoxic or environmental stimuli. Another limitation was that tumor core regions were analyzed, while tumor margins in the invasive zone were not investigated. However, other studies have pointed to the importance of core regions in tumor progression due to silencing/methylation as a consequence of tissue hypoxia [33].

In conclusion, our study provides the first map of apoptosis sensitivity at individual protein and systems level in intact CRC tissue. We holistically describe both patient-to-patient and intra-tumor heterogeneity in apoptosis signaling in stroma, immune, and cancer cells that have important implications for the future use of apoptosis sensitizers in the treatment of CRC.

## Materials and methods

### Materials

Sources of antibodies, cell lines, and software are all listed in Supplementary Table 1.

### Colorectal cancer cohort

Formalin-fixed, paraffin-embedded primary tumor tissue sections were obtained from 170 chemotherapy-naïve, resected stage III CRC patients. Tumor samples were collected from three centers: Beaumont Hospital (RCSI, Ireland), Queen’s University Belfast (UK), and Paris Descartes University (France). A summary of the clinical characteristics of the cohort is provided in Supplementary Table 2. Data of 46 cores of 36 patients were dropped after quality assessment of the stained tissue (see below).

### Cell lines

Three technical replicates (cores) of pellets of formalin-fixed HeLa, Jurkat, MCF7, SKMEL, HCT-116 SMAC^KO^, and HCT-116 XIAP^KO^ cells in which quantities of mitochondrial apoptosis proteins were previously determined [2, 10, 34] were included in the construction of the tissue microarray (TMA) in parallel to the patients’ cores, and served as quality control and internal standards for protein quantification. Three of 18 cores of two cell lines were removed after quality control. Cells were grown to 80% confluence. Media was replaced 12–24 h before fixation. To fix cells, cells were gently washed in sterile 1XPBS solution. Cell monolayers were covered with 5 mL 10% neutral-buffered formalin (NBF) for 2–5 min. Cells were scraped into NBF, and collected into labeled 50 mL tubes, and stored at 4 °C for at least 3–4 h. For further processing, cells were centrifuged at 1200 rpm for 5 min and washed in 1% low melt agarose solution XBPS before re-suspension in 0.5 mL 80% ethanol and centrifugation at 12,000 rpm twice for 5 min. Subsequently, 80% of ethanol was aspirated and cell pellets were molded into caps and frozen, prior to TMA construction.

### Antibody validation and conjugation

Commercially acquired antibodies underwent multi-step process of validation and conjugation (as previously described by Gerdes et al. [19]). Briefly, at least two to three clones for each target were stained in parallel using a multi-tissue array (MTU 481, Pantomics, CA) and staining performance visually compared. At least one antibody clone was downselected for conjugation with either Cy3 or Cy5 bis-NHS-ester dyes. Epitopes were also tested for sensitivity to the dye-inactivation solution (basic hydrogen peroxide) by exposing multi-tissue arrays to 0, 1, and 10 rounds the solution and stained with the antibody of interest and compared. Approximately 10% of epitopes have been shown to have decreased signal following exposure to the inactivation solution and those antibodies are placed early in the multiplexing sequence [19].

### Immunofluorescence imaging of patient TMAs

Multiplexed immunofluorescence iterative staining of the CRC TMAs was performed as previously described [19] using the Cell DIVE™ technology (Cytiva, Issaquah, WA; formerly GE Healthcare). This involves iterative staining and imaging of the same tissue section with 60+ antibodies and is achieved by mild dye oxidation between successive staining and imaging rounds. In total, there were 13 staining rounds using the antibodies described above and DAPI was imaged in each round. The Leica Bond (Leica Biosystems) was used for antibody staining and the IN Cell 2200 was used for imaging. Staining and image recording was repeated twice for S6 due to staining failure. Exposure times were set to fixed values for all images of a given marker.

### Image pre-processing and quality control

Immunofluorescent images were processed and cells were segmented and quantified as described previously [19, 35]. A total of 48 positions showing major cell loss during staining rounds were excluded from all analysis, as well as cells within the images’ margins of 15 pixel on the *x*-axis and 10 pixel on the *y*-axes were dropped from all data analysis. A total of 74 positions showing major or minor cell loss during staining rounds were excluded from training datasets for post-processing such as batch correction or cell classification. For QC analysis, we extended the method presented in Bello and we generated automated QC scores [36] (0–1) for every cell in each imaging round by correlating baseline DAPI images with all corresponding DAPI images from other multiplexing rounds. Cells included in the analysis had a median QC score of 0.95, with 53% having a QC score greater than 0.8. The average QC score was 0.57. In comparison, 83% of cells removed during quality control had a QC score less than 0.1 with an average QC score of 0.15. Following quantification, slides were normalized for batch effects and exposure time for each channel/marker analyzed.

### Post pre-processing and batch correction

To correct for a possible batch effects between slides, cells’ mean intensity were first normalized using upper-quantile normalization, grouped by protein marker and slide. Secondly, quantiles of the normalized intensities were plotted against their rankits, and an affine transformation matrices to rotate the function to the main diagonal were calculated in regulator and helper T cells. Obtained transformation matrices were applied on the intensities, and pixel intensity values were restored using linear regression and upper-quantile normalized values. Solely for the batch correction, cells within 5% of the images’ margins were excluded for the calculation of the reference values. The batch correction was quality controlled with cell lines spotted in parallel to tissue samples on three of five slides.

### Immune cell classification

To differentiate cell types, we used CD3, CD4, CD8, CD45, FOXP3, PCK26, and Cytokeratin AE1 markers. We manually annotated 4839 AE1- or PCK27-positive cells as (epithelial) cancer cells. Of 3121 CD3-positive cells [37], 788 CD4-positive cells were annotated as helper T cells [37], 991 CD8-positive cells were annotated as cytotoxic T cells [37], and 1360 FOXP3-positive cells were annotated as regulatory T cells [38]. A total of 3369 CD3-negative cells that were either CD4, CD45, or CD8 positive were annotated as other leukocytes. In total, 3837 cells lacking any marker (but were DAPI) positive were annotated as stroma-rich cells (other stromal cells). Using the manual annotations, we constructed a random forest of 2000 trees (R package randomForest, version 4.6-14) and employed it to classify all cells. Regulator and helper T cells annotation were used for separate slide depended models used to define the reference population for the batch correction using affine transformation matrices.

### Protein profiling and apoptosis sensitivity modeling

Protein levels of BAK, BAX, BCL2, BCL(X)L, and MCL1 were normalized to the mean protein levels in HeLa cells spotted in parallel to patients’ core on three of five slides. Protein’s molar concentrations were calculated using previously established HeLa concentrations and used as input for DR_MOMP as previously described [2]. DR_MOMP [2] was translated from its MATLAB implementation to C++ and R using deSolve (1.28), doParallel (1.0.15), and Rcpp (1.0.5). For each core the maximum % level of pores was calculated after simulating an approximated mean stress dose (200 nM; estimated from Fig. 4B 191 nM) of the patient population as threshold [2, 4, 39]. Cells were considered to have low sensitivity for MOMP if the % level of pores was <10% [2]. We found a Pearson correlation coefficient of −0.82 (95% CI −0.83 to −0.82; *p* < 0.001) between the % level of pores and the (previously) used stress dose required for MOMP [2] (log-transformed) calculated for 10,000 randomly chosen cancer cells.

APOPTO-CELL [10] was executed in MATLAB with the Statistics and Parallel toolboxes (version 2014b, The MathWorks, Inc., Natick, MA, USA). Molar protein concentrations for the APOPTO-CELL input proteins PRO-CASPASE 3, PRO-CASPASE 9, SMAC, and XIAP were estimated by aligning signal intensities [a.U.] to profiles [µM] determined in a reference clinically relevant CRC cohort [20] with an established pipeline [3] and protein molar concentrations of a reference CRC cohort [20]. Previous research [16, 20] has shown APAF1 to be the limiting factor in apoptosome formation in the CRC settings [16, 20] and was set to 0.123 µM as described previosuly [3, 16].

For both models, we performed two sets of simulations: (1) per-core and (2) per-cell. For the per-core simulations, we aggregated (by median) the batch-corrected protein intensities across all cells for each core per patient prior to conversion to molar concentrations, resulting in one simulation per core and thus two to three simulations per patient. For the per-cell simulations, we performed a simulation for each cell, totaling ~3.5 million simulations for 168 patients included in the study.

### Statistical analysis

All statistical tests were performed in R (3.6.3) and *p* values <0.05 were considered statistically significant. All data are presented as mean ± SEM. All statistical tests were performed in R. If not otherwise mentioned, two-tailed *t* tests were performed for pairwise comparison, while ANOVAs with Tukey honest significance post-hoc tests were performed in cases of the comparison of three or more populations. The quartile COD were calculated using (Q_3_ – Q_1_) / (Q_3_ + Q_1_) with Q_*n*_ be the respective quartiles. Shannon Entropy was calculated either using log_2_ for binary populations or the natural logarithm, with 10^−10^ added to all values. Moran’s I was calculated using the R package ape (5.4-1) without outliers and only on populations >100 cells. Distances >2000 px were set to 2000 px. Consensus clustering was performed using ConsensusClusterPlus (1.48.0) with a seed of 42, 100,000 repetitions, Spearman and Ward’s method as parameters. For the bootstrap analysis, slides were 100,000 times randomly paired using a seed of 42. A Cox proportional hazard regression analysis was performed in all stage III patients for which three cores were available (*n* = 36 patients). Models included mean protein levels or APOPTO-CELL readouts, Shannon entropy, or Moran’s I score, and calculated their interaction using all available cores (*n* = 108). Models were adjusted for age and gender. For the interaction term only, *p* < 0.1 was considered to be statistically significant.

## Supplementary information

Supplementary Table and Supplementary Figure legends

Supplementary Figure 1

Supplementary Figure 2

Supplementary Figure 3

Supplementary Figure 4

Supplementary Figure 5

Supplementary Figure 6

Supplementary Figure 7

Supplementary Table 1

Supplementary Table 2

Supplementary Table 3

Supplementary Table 4

## Data Availability

Imaging data, cell masks, and generated single-cell measurements of 20 markers are available from the lead contact. Further information and request for code or resources should be directed to and will be fulfilled by the lead contact.
