# Supplementary material for: An atlas of inter- and intra-tumor heterogeneity of apoptosis competency in colorectal cancer tissue at single-cell resolution
Source: Cell Death Differ. 2021 Nov 9;29(4):806–17. doi: 10.1038/s41418-021-00895-9 (PMC8990071; doi:10.1038/s41418-021-00895-9)
Supplement: Supplementary file 1 — Supplementary Table and Supplementary Figure legends [file 41418_2021_895_MOESM1_ESM.docx]

An atlas of inter- and intra-tumor heterogeneity of apoptosis competency in colorectal cancer tissue at single cell resolution

## *Expanded View Table and Figure legends*

Andreas Ulrich Lindner^1,2^, Manuela Salvucci^1,2^, Elizabeth McDonough^3^, Sanghee Cho^3^, Xanthi Stachtea^4^, Emer P O’Connell^1,2,5^, Alex D Corwin^3^, Alberto Santamaria-Pang^3^, Steven Carberry^1,2^, Michael Fichtner^1,2^, Sandra Van Schaeybroeck^4^, Pierre Laurent-Puig^6^, John P Burke^5^, Deborah A McNamara^5,7^, Mark Lawler^4^, Anup Sood^3^, John F Graf^3^, Markus Rehm^8^, Philip D Dunne^4^, Daniel B Longley^4^, Fiona Ginty^3^, Jochen HM Prehn*^1,2^

^1^Department of Physiology and Medical Physics; ^2^Centre of Systems Medicine; ^5^Department of Surgery, Royal College of Surgeons in Ireland University of Medicine and Health Sciences, 123 St. Stephen’s Green, Dublin 2, Ireland.
^3^GE Research, Niskayuna, NY 12309, USA.
^4^Centre for Cancer Research & Cell Biology, Queen’s University Belfast, 97 Lisburn Road, Belfast, BT9 7AE, Northern Ireland, UK.
^6^Centre de Recherche des Cordeliers, INSERM, CNRS, Université de Paris, Sorbonne Université, USPC, Equipe labellisée Ligue Nationale Contre le Cancer, Paris, France.
^7^Beaumont Hospital, Beaumont Road, Dublin 9, Ireland
^8^Institute of Cell Biology and Immunology, University of Stuttgart, Allmandring 31, 70569 Stuttgart, Germany.

***Corresponding Author:** Jochen HM Prehn, Department of Physiology and Medical Physics, Royal College of Surgeons in Ireland, 123 St. Stephen’s Green, Dublin 2, Ireland; prehn@rcsi.ie.

# Expanded View Table and Figure legends

Supplementary Table 1 – key resource table.

Supplementary Table 2 – Patient information with mean cell fractions and DR_MOMP and APOPTO-CELL results for aggregated protein levels for patient-matched cores.

Supplementary Table 3 - Transcriptional data derived from flow-sorted immune, epithelial and fibroblast populations isolated from CRC primary tumor tissue (GSE39396).

Supplementary Table 4 – Result table for the multivariate Cox proportional Hazard Regression analysis of the effect of the Shannon entropy and Moran’s I, together with the respective mean protein levels or APOPTO-CELL model predictions, and the effect of the interaction of both terms in cancer cells on patient’s disease free survival (Suppl. Figure 7).

Supplementary Figure 1 - Plot of patients’ consensus cluster score of patient-matched cores after hierarchical consensus clustering using cancer, immune and stroma cell fractions of each core. Patients with a low consensus score (0) show high difference in cell fractions between matched cores while patients with a high consensus score (1) show high similarity in cell fractions between matched cores.

Supplementary Figure 2 – Box plot of transcriptional data derived from flow-sorted immune (n = 6), epithelial (n = 6) and fibroblast (n = 6) populations isolated from CRC primary tumor tissue (GSE39396^42^; Suppl. Table 2; ANOVA and Tukey post-hoc).

Supplementary Figure 3 - Box plot of quartile coefficients of dispersion of protein levels of each core and stratified for cancer (red), immune (blue) and stroma cells (grey).

Supplementary Figure 4 - In analog to the correlation plot in Figure 3E-G showing the median correlation coefficient in all (black), cancer (red), immune (blue) and stroma (gray) cells, but including the interquartile range.

Supplementary Figure 5 - Calculated the Shannon Entropy for the proteins using bins for protein level with a bin width of z-score = 0.1 SD for each protein respectively and stratified for cancer (red), immune (blue) and stroma (gray) cells. Proteins were sorted base for (A) DR_MOMP and (B) APOPTO-CELL.

Supplementary Figure 6 - Calculated cores’ Moran’s I for low/high (I) sensitivity for MOMP for protein levels stratified for cancer (red), immune (blue) and stroma (gray) cells. Proteins were sorted base for (A) DR_MOMP and (B) APOPTO-CELL.

Supplementary Figure 7 - Forest plot showing multivariate Cox proportional Hazard Regression analysis of the effect of (AB) the Shannon entropy and (CD) Moran’s I, together with the respective (AC) mean protein levels or (BD) APOPTO-CELL predicted caspase activity (calculated substrate cleavage), and the effect of the interaction of both terms in cancer cells on patient’s disease free survival. Hazard ratios (HR) were depicted numerically. Models were adjusted for age and gender. Only stage III patients with three matched cores were included. We considered an alpha of 0.1 instead 0.05 for the interaction term. Plotted HR is log10-transformed with 95% confidence interval displayed as range. NS indicates a not significant p ≥ 0.1 for the interaction term. Full results are presented in Supplementary Table 4.
