# Supplementary figures and images for: An atlas of inter- and intra-tumor heterogeneity of apoptosis competency in colorectal cancer tissue at single-cell resolution
Source: Cell Death Differ. 2021 Nov 9;29(4):806–17. doi: 10.1038/s41418-021-00895-9 (PMC8990071; doi:10.1038/s41418-021-00895-9)

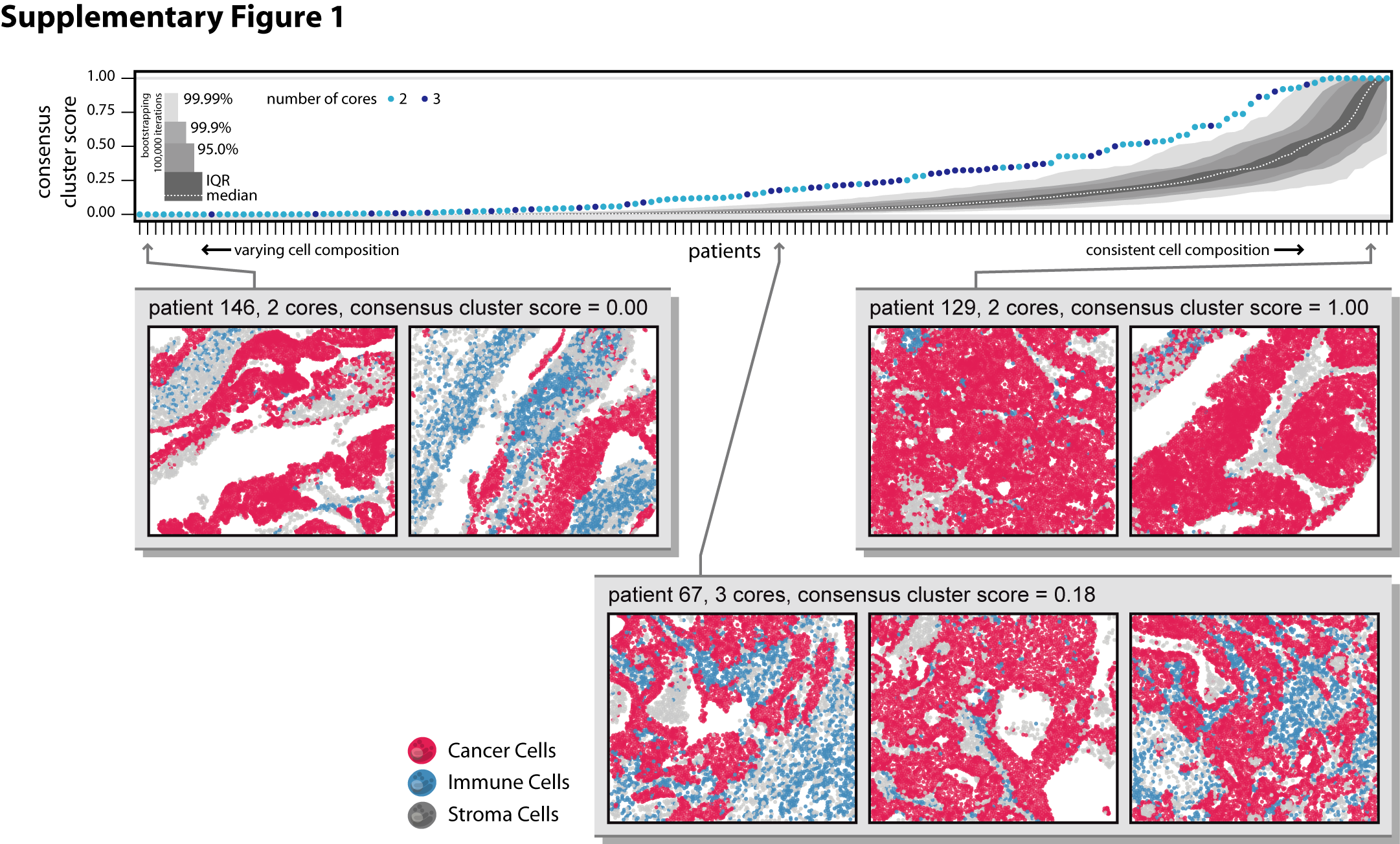

Supplement: Supplementary file 2 — Supplementary Figure 1 [file 41418_2021_895_MOESM2_ESM.png]

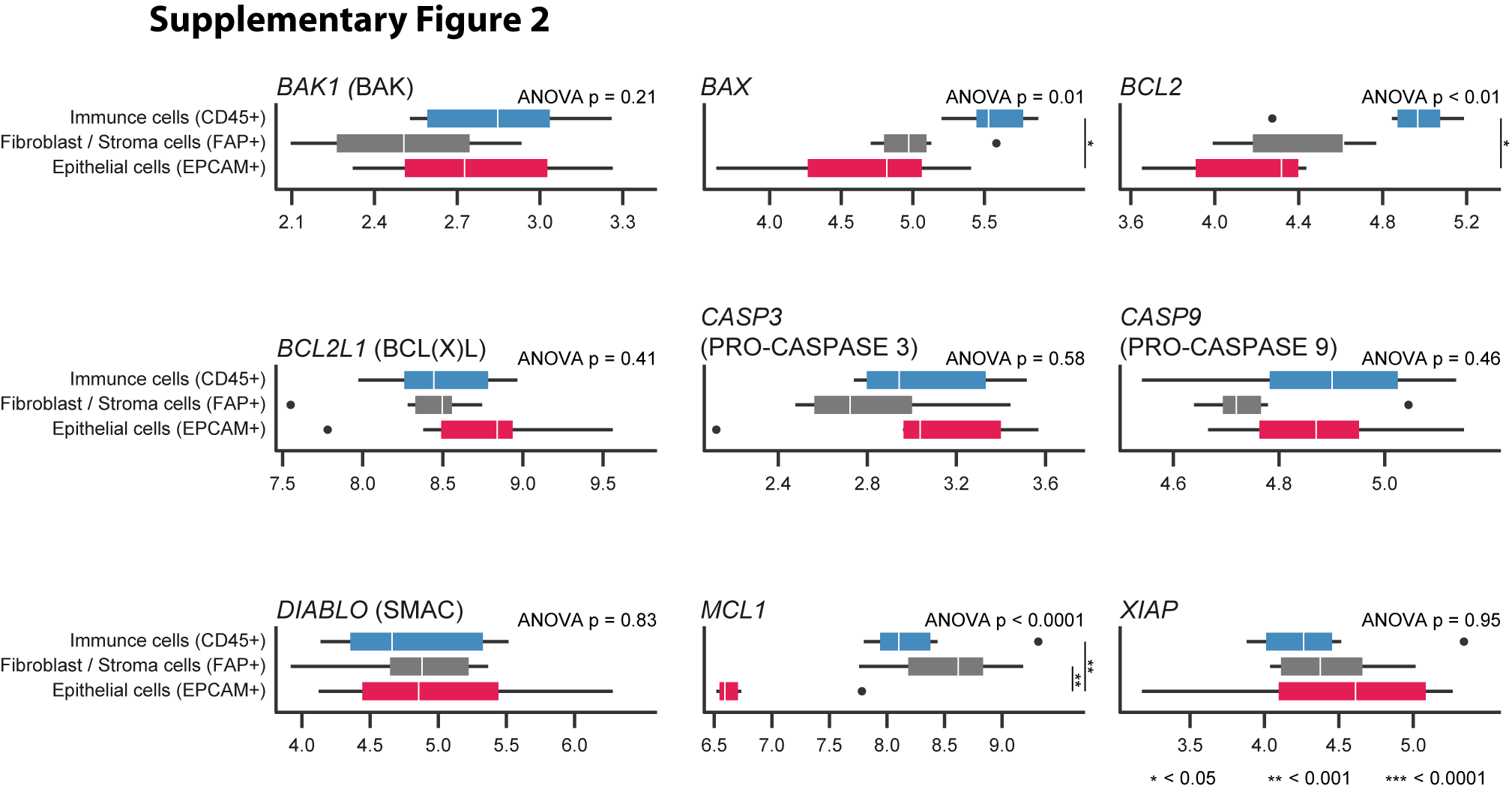

Supplement: Supplementary file 3 — Supplementary Figure 2 [file 41418_2021_895_MOESM3_ESM.png]

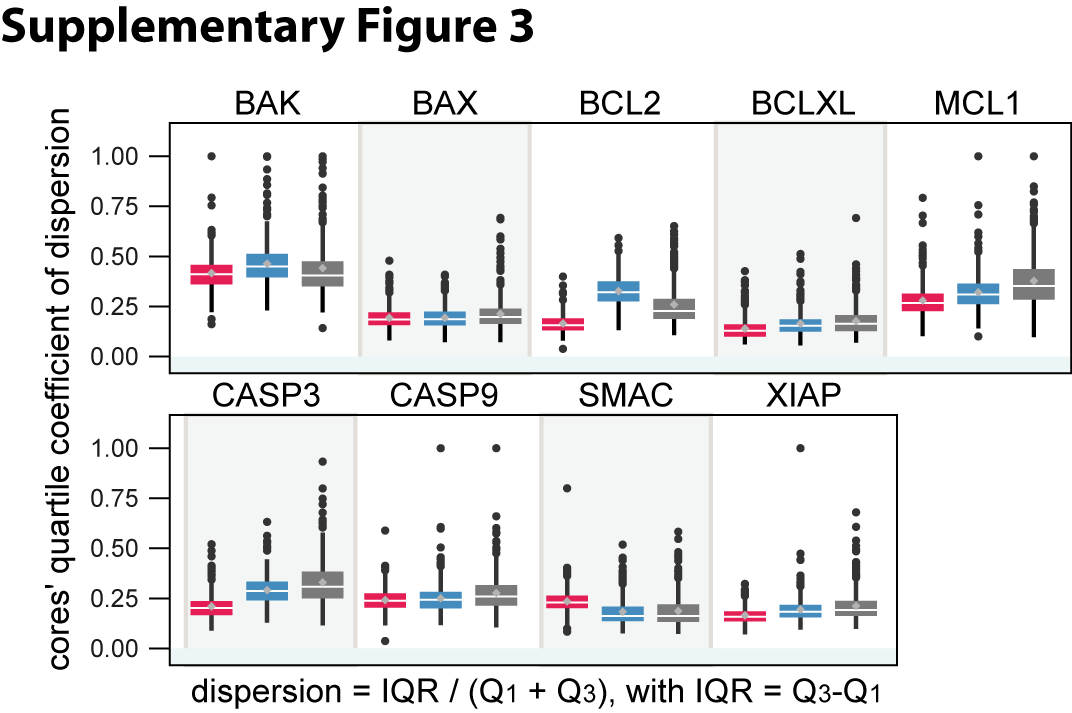

Supplement: Supplementary file 4 — Supplementary Figure 3 [file 41418_2021_895_MOESM4_ESM.png]

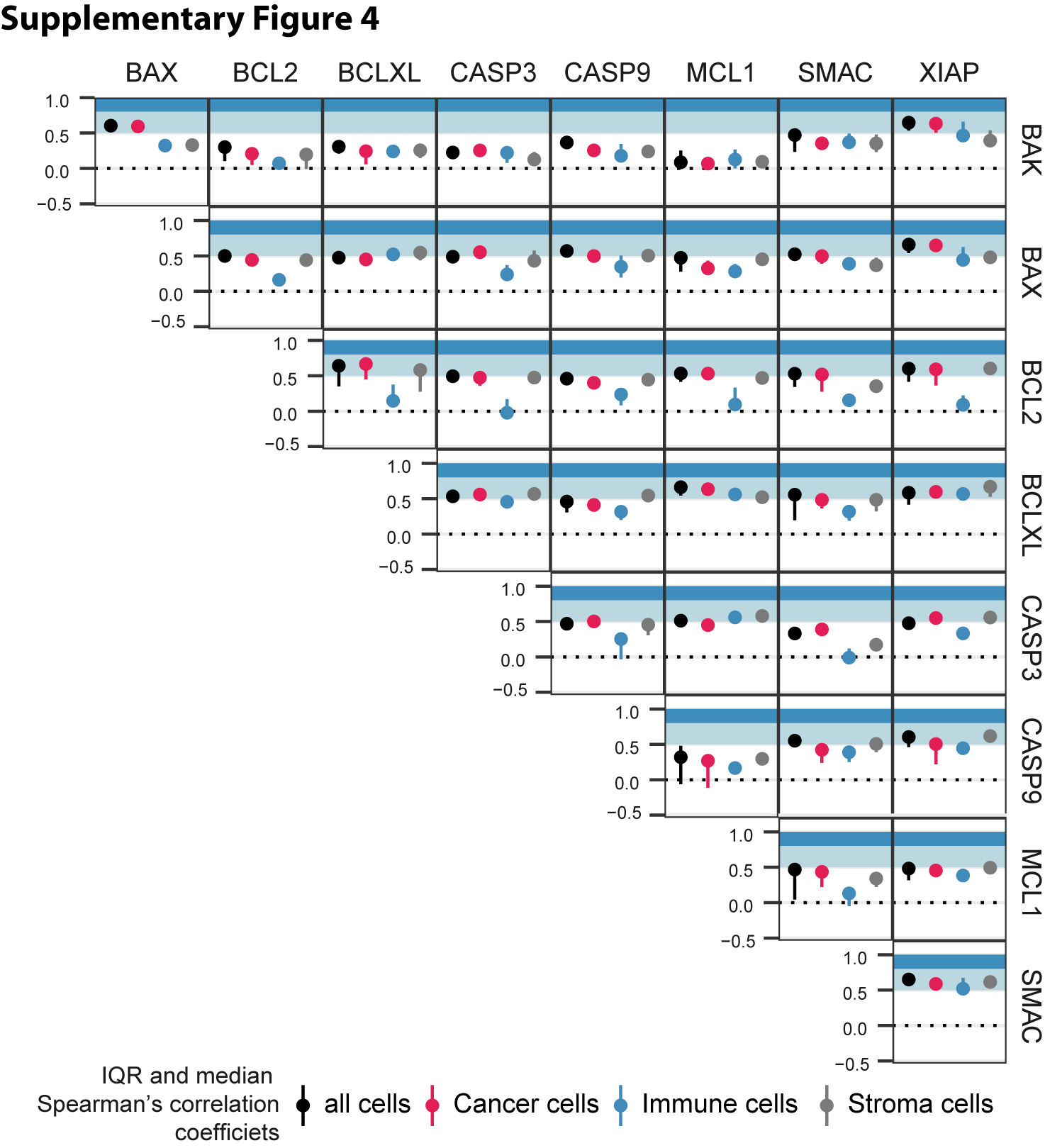

Supplement: Supplementary file 5 — Supplementary Figure 4 [file 41418_2021_895_MOESM5_ESM.png]

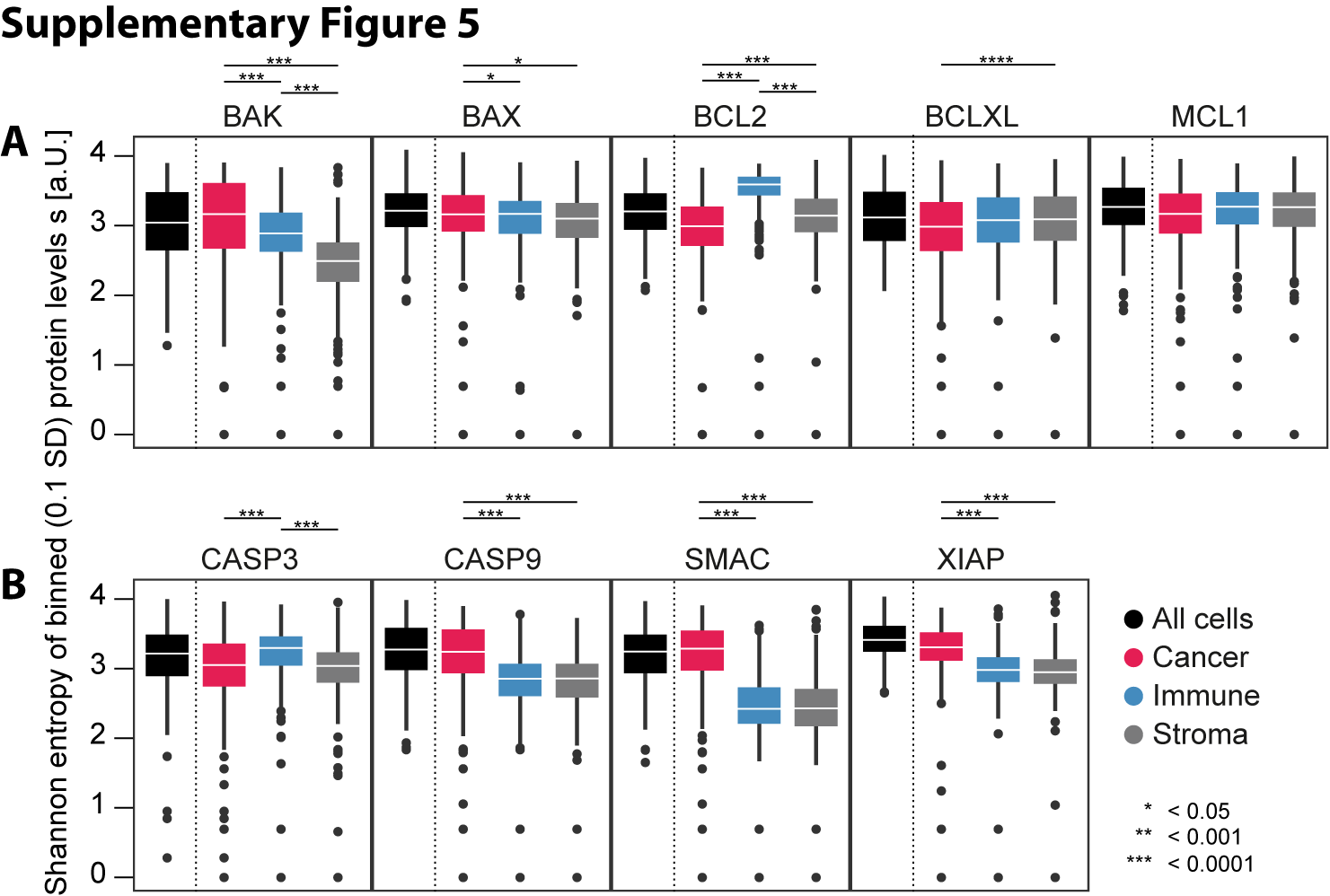

Supplement: Supplementary file 6 — Supplementary Figure 5 [file 41418_2021_895_MOESM6_ESM.png]

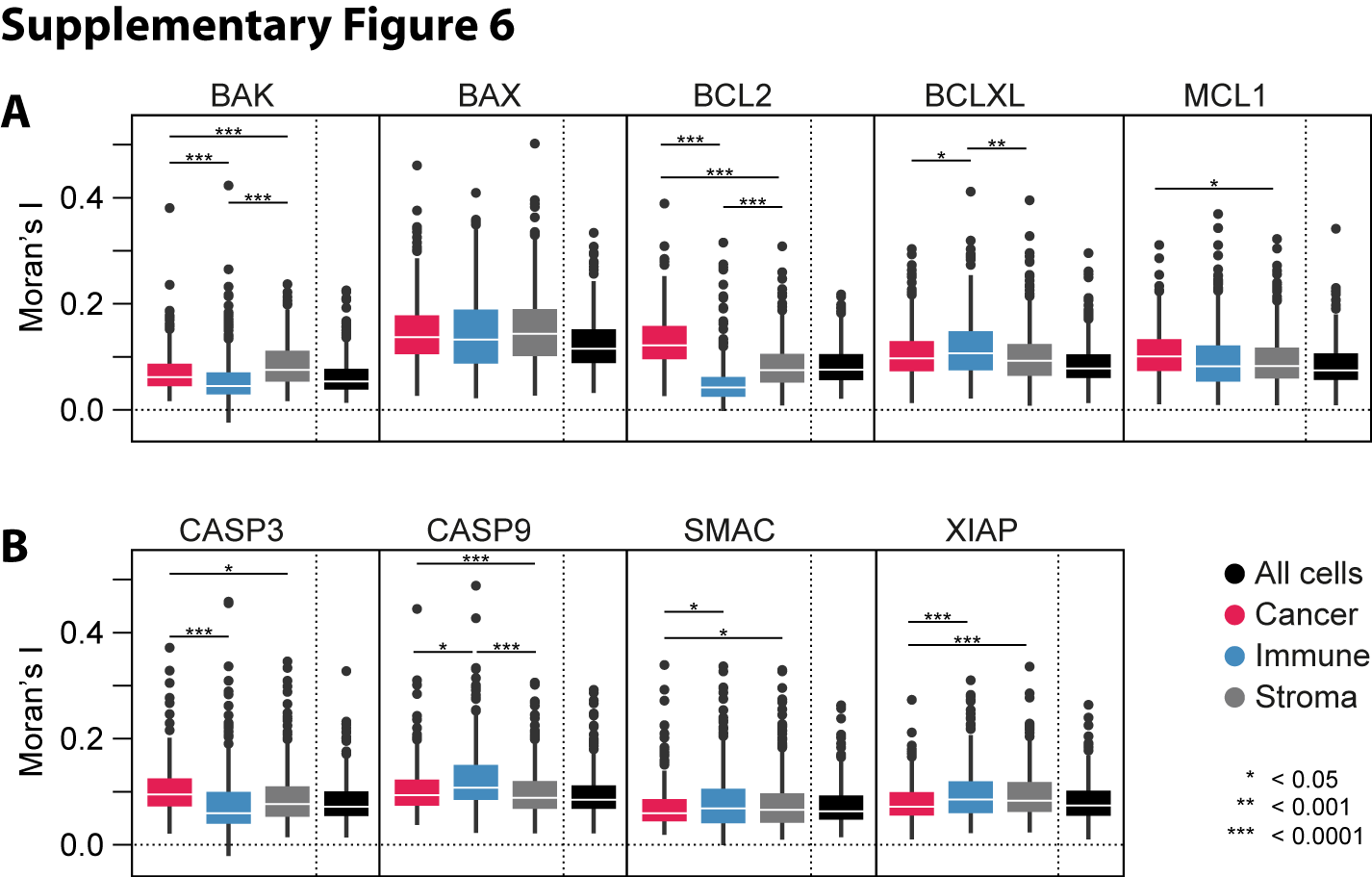

Supplement: Supplementary file 7 — Supplementary Figure 6 [file 41418_2021_895_MOESM7_ESM.png]

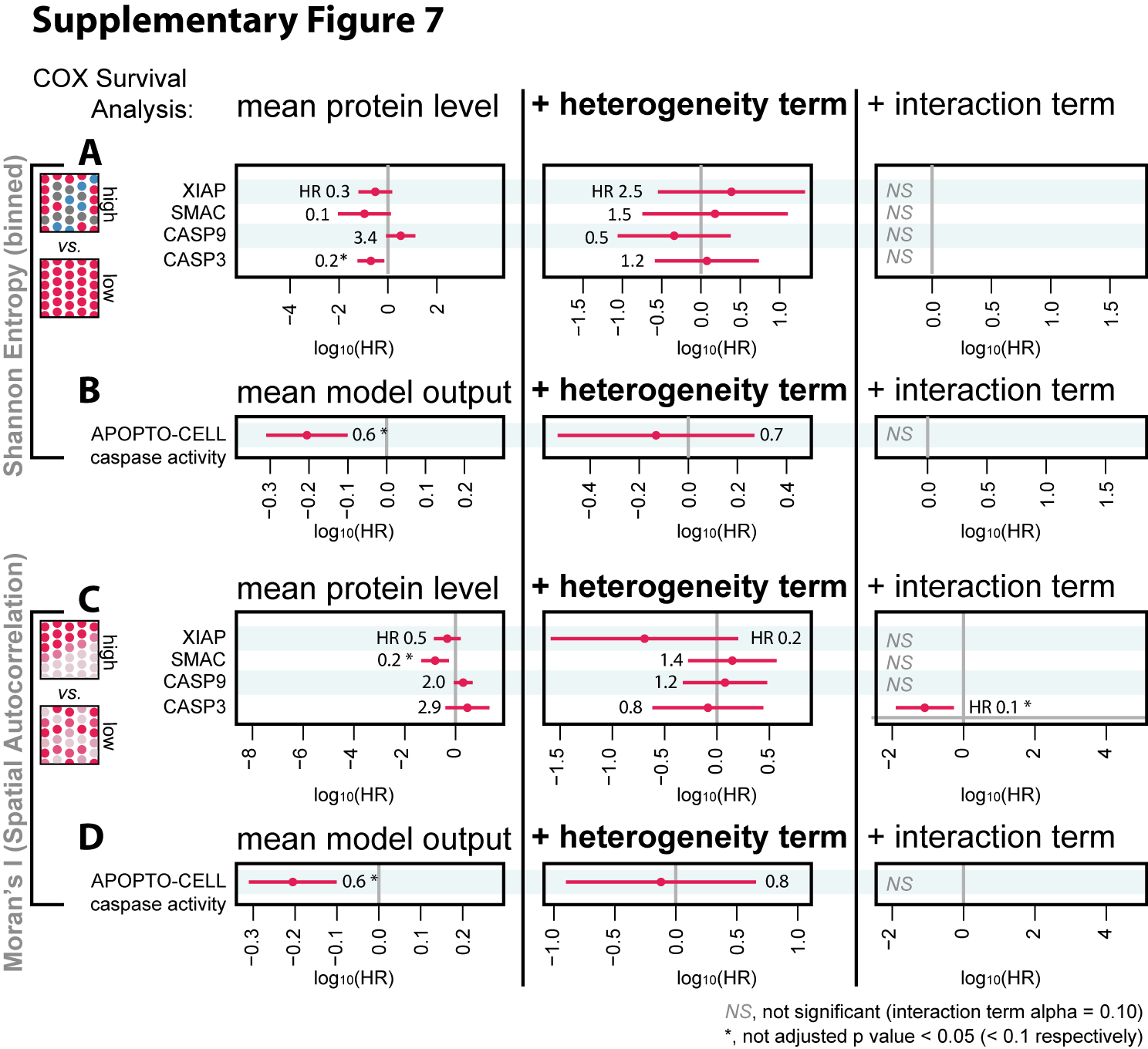

Supplement: Supplementary file 8 — Supplementary Figure 7 [file 41418_2021_895_MOESM8_ESM.png]
