# Supplementary material for: An atlas of inter- and intra-tumor heterogeneity of apoptosis competency in colorectal cancer tissue at single-cell resolution
Source: Cell Death Differ. 2021 Nov 9;29(4):806–17. doi: 10.1038/s41418-021-00895-9 (PMC8990071; doi:10.1038/s41418-021-00895-9)
Supplement: Supplementary file 9 — Supplementary Table 1 [file 41418_2021_895_MOESM9_ESM.docx]

## Supplementary Table 1

| REAGENT or RESEOURCE | SOURCE | IDENTIFIER or CONTACT | |  |  |
| --- | --- | --- | --- | --- | --- |
| **Antibodies** | | | | | |
| APAF-1 | Millipore (Burlington, MA, USA) | 2E12 | MAB3053 |  |  |
| Bak | Cell Signaling (Danvers, MA, USA) | D4E4 | 12105 |  |  |
| Bax | Abcam (Cambridge, UK) | E63 | ab216985 |  |  |
| BCL-2 | LifeSpan Bioscience (Seattle, WA, USA) | 124 | LS-C389442 |  |  |
| Bcl-xL | Thermo Fisher (Waltham, MA, USA) | 7D9 | MS-1334 |  |  |
| CA9 | Thermo Fisher | polyclonal | PA1-16592 |  |  |
| Caspase-3 | Cell Signaling | D3R6Y | 14214 |  |  |
| Caspase-9 | Santa Cruz (Dallas, TX, USA) | 96.1.23 | sc-56076 A647 |  |  |
| CD3 | Dako (Santa Clara, CA, USA) | F7.2.38 | M7254 |  |  |
| CD4 | Abcam | EPR6855 | ab181724 |  |  |
| CD8 | Dako | C8/144B | M7103 |  |  |
| CD45 | Dako | 2B11 + PD7/26 | M0701 |  |  |
| Cytokeratin AE1 | eBioscience (San Diego, CA, USA) | AE1 | 14-9001 |  |  |
| Cytokeratin PCK26 | Sigma (St. Louis, MO, USA) | PCK26 | C1801 |  |  |
| FOXP3 | Biolegend (San Diego, CA, USA) | 206D | 320014 |  |  |
| Glut-1 | Abcam | EPR3915 | ab196357 |  |  |
| HLA I | Abcam | EMR8 5 | ab70328 |  |  |
| Ki67 | Zeta (Sierra Madre, CA, USA) | SP6 | Z2031 |  |  |
| MCL-1 | Abcam | Y37 | ab186822 |  |  |
| NAKATPase | Abcam | EP1845Y | ab167390 |  |  |
| S6 | Santa Cruz | C-8 | sc-74459 A647 |  |  |
| Smac | Cell Signaling | 79-1-83 | 2954 |  |  |
| PD1 | Abcam | EPR4877(2) | ab201825 |  |  |
| XIAP (API3) | Thermo Fisher | polyclonal | APH937 |  |  |
| **Cell Lines** | | | | | |
| HCT-116 SMAC KO | Dr. B Vogelstein (John Hopkins University, MD, USA) | | | |  |
| HCT-116 XIAP KO | Dr. B Vogelstein (John Hopkins University, MD, USA) | | | |  |
| HeLa | American Type Culture Collection (LGC Standards) | | | |  |
| JURKAT | Dr. PH Krammer and Dr. H Walczak (DKFZ, DE) | | | |  |
| MCF7 | Dr. RU Jänicke (University of Düsseldorf, DE) | | | |  |
| SKMEL | DSMZ, Germany | ACC 151 | |  |  |
| **Biological Samples** | | | | | |
| Stage III primary CRC tumour tissue resect prior 5-FU based chemotherapy | Beaumont Hospital (RCSI, IE) | *NA* | |  |  |
|  | Queen’s University Belfast (UK) | *NA* | |  |  |
|  | Paris Descartes University (FR) | *NA* | |  |  |
| **Cell DIVE Platform** | | | | | |
| Cell DIVE™ | Cytiva; GE Research | fiona.ginty@ge.com | |  |  |
| Multi tumor tissue array | Pantomics | MTU 481 | |  |  |
| **Software and algorithms** | | | | | |
| R (3.6.3) | R Foundation | www.r-project.org | |  |  |
| Fiji (ImageJ; 1.51k) | Schindelin *et al.*^1^ | www.fiji.sc | |  |  |
| GE SingleCellMetrics Plugin | GE Research | fiona.ginty@ge.com | |  |  |
| Layers cell analysis software version 1 | GE Research | fiona.ginty@ge.com | |  |  |
| APOPTO-CELL | Rehm and Huber *et al.*^2, 3^ | prehn@rcsi.ie | |  |  |
| DR_MOMP | Lindner *et al.*^4^ | prehn@rcsi.ie | |  |  |
| MATLAB with the Statistics and Parallel toolboxes (version 2014b) | The MathWorks | www.mathworks.com | |  |  |

# References

1. Schindelin J, Arganda-Carreras I, Frise E, Kaynig V, Longair M, Pietzsch T*, et al.* Fiji: an open-source platform for biological-image analysis. *Nat Methods* 2012, **9**(7)**:** 676-682.

2. Rehm M, Huber HJ, Dussmann H, Prehn JH. Systems analysis of effector caspase activation and its control by X-linked inhibitor of apoptosis protein. *EMBO J* 2006, **25**(18)**:** 4338-4349.

3. Huber HJ, Rehm M, Plchut M, Dussmann H, Prehn JH. APOPTO-CELL--a simulation tool and interactive database for analyzing cellular susceptibility to apoptosis. *Bioinformatics* 2007, **23**(5)**:** 648-650.

4. Lindner AU, Concannon CG, Boukes GJ, Cannon MD, Llambi F, Ryan D*, et al.* Systems analysis of BCL2 protein family interactions establishes a model to predict responses to chemotherapy. *Cancer Res* 2013, **73**(2)**:** 519-528.
